# An atypically located large subchondral cyst in an osteoarthritic hip joint: a case report

**DOI:** 10.1186/1752-1947-7-176

**Published:** 2013-07-05

**Authors:** Melih Güven, Turhan Özler, Onur Kocadal, Ferda Özkan, Faik Altıntaş

**Affiliations:** 1 Department of Orthopaedics and Traumatology, Yeditepe University School of Medicine, Istanbul, Turkey; 2 Department of Pathology, Yeditepe University School of Medicine, Istanbul, Turkey

**Keywords:** Hip, Osteoarthritis, Soft tissue, Subchondral cyst, Tumor

## Abstract

**Introduction:**

Osteoarthritic subchondral cysts within or around the hip joint can sometimes be difficult to distinguish from primary osseous and soft tissue tumors due to their radiological appearance and uncommon location.

**Case presentation:**

We report the case of a 74-year-old Turkish man with a subchondral cyst arising from the hip joint, eroding the acetabulum and located on the medial side of the iliac bone, which imitated a soft tissue tumor. This cystic lesion was resected and the results of histopathological analysis of tissue samples were found to be consistent with an osteoarthritic cyst.

**Conclusions:**

The present case illustrates how an osteoarthritic subchondral cyst can grow into the soft tissue planes in the presence of destruction of the articular cartilage and subchondral bone continuity, and present as an apparent soft tissue tumor.

## Introduction

Osteoarthritis (OA) is a degenerative joint disease with some radiological characteristics including the presence of multiple, small cysts (geodes) with subchondral sclerosis, marginal osteophytes, intra-articular osteochondral bodies and narrowing or loss of joint space [1,2]. Osteoarthritic subchondral cysts frequently occur in weight-bearing joints such as the hip. The etiology of these cysts in hip OA remains uncertain. Theories on their pathogenesis include that they originate from intrusion of synovial fluid into the bone at the joint surface, initiate in areas of bone necrosis, or are confined to pressure segments in the femoral head and acetabulum [3-8]. Regardless of their etiology, subchondral cysts are generally thought to develop in bone adjacent to highly degenerated joint surfaces and, as a result, are frequently found in weight-bearing areas of the osteoarthritic hip joint at the time of total hip arthroplasty [8]. However, large symptomatic subchondral cysts in the hip joint can sometimes be difficult to distinguish from primary osseous and soft tissue tumors due to their radiological appearance and uncommon location [9-11].

In the present work, we report the case of a patient with an atypically located osteoarthritic cyst arising from the hip joint, eroding the acetabulum and located on the medial side of the iliac bone, which imitated a soft tissue tumor on radiological imaging studies.

## Case presentation

A 74-year-old Turkish man was referred to our out-patient clinic because of right hip pain without a history of trauma. He had experienced severe pain in the groin on weight bearing, with variable degree of pain at rest, over the last 45 days. He was able to walk only with the assistance of crutches. His medical history was unremarkable.

On physical examination, he was afebrile and had a blood pressure of 120/80mmHg. His active ranges of motion (ROM) for both hips were restricted in all directions and the passive ROM of right hip was painful in flexion, abduction and internal rotation. The result of a Thomas test was positive for the right hip.

**Figure 1:**
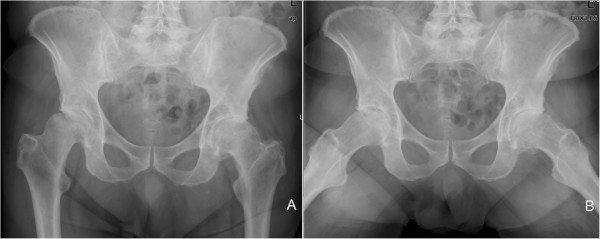
Antero-posterior (A) and frog-leg (B) pelvis radiographs showing sclerotic changes in both acetabulum, loss of articular joint distance, osteophytes and degenerative changes in both hips, which indicate severe degenerative coxarthrosis.

**Figure 2:**
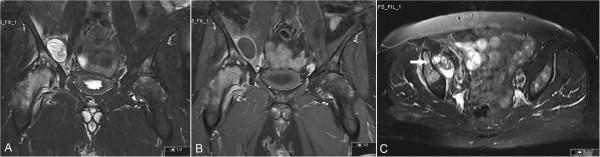
**Coronal (A and B) and axial (C) magnetic resonance imaging scans.** Coronal T2-weighted fat-saturated (**A**) and T1-weighted fat-saturated contrast-enhanced (**B**) magnetic resonance imaging scans showing a cystic lesion located on the medial side of the right iliac bone. The cyst is associated with the adjacent acetabulum (white arrow) on the axial fat-saturated magnetic resonance imaging scan (**C**).

Antero-posterior and frog-leg pelvis radiographs (Figure 1) showed lucent and sclerotic regions in both the femur and acetabulum with loss of articular joint distance and flattening especially in the right femoral head, which indicated severe degenerative coxarthrosis. Magnetic resonance imaging (MRI) scans revealed a cystic lesion located on the medial side of the iliac bone, which had a uniformly bounded capsule and contained serpentine-like structures (Figure 2A,B). On an axial MRI image (Figure 2C), the cystic lesion was seen to reach antero-medially to the hip joint and had eroded the adjacent acetabulum. The results of an abdomino-pelvic ultrasound indicated a grade I hepatosteatosis and 40mm calcified cortical cyst located on the upper pole of the right kidney. The results of standard laboratory tests revealed a normal level of hemoglobin (13.1g/dL), hematocrit (38) and white blood cell (6.48mm^3^/μL) counts. The results of functional tests for the kidney and liver, as well as other biochemical blood analyses, were normal.

Surgery for excision of the cystic lesion was recommended, and was performed under general anesthesia. Our patient lay in a supine position and an anterior ilioinguinal incision was made in the right hip. The interval between the tensor fascia lata and sartorius muscle was identified. The lateral femoral cutaneous nerve was retracted laterally. The dissection was extended proximally to expose the medial surface of the iliac bone. The rectus femoris muscle was not incised from its attachment to the upper part of the acetabular rim, but was instead retracted laterally. The iliacus muscle was identified and stripped from the medial surface of the iliac bone. The cystic lesion was identified. There were no adhesions between the cyst membrane and surrounding soft tissue. However, it was associated with the antero-medial acetabular wall and had eroded the adjacent acetabulum. The cystic lesion was resected en bloc and examined in the operating room on the surgical table. Calcified necrotic material was exposed in the cyst.

**Figure 3:**
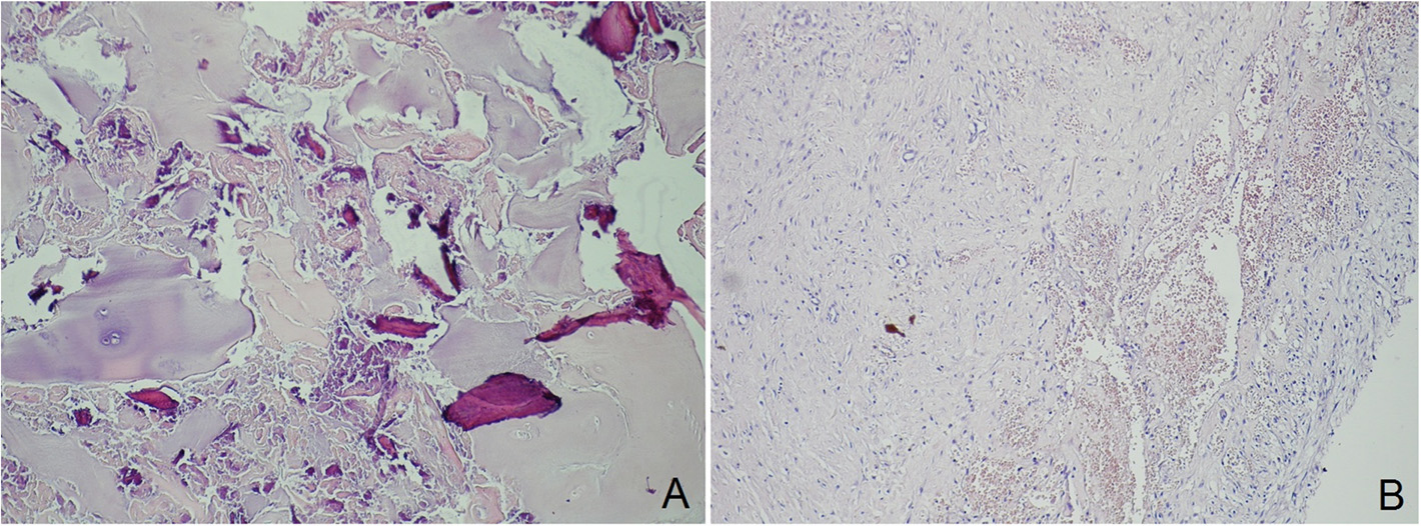
**Histopathologic investigation of the cystic lesion (A) and cyst membrane (B).** Histopathologic investigation of the cystic lesion (**A**) shows necrotic bone trabeculae and chondroid tissue (hematoxylin and eosin stain, ×400). Histopathological view of the cyst membrane (**B**) (hematoxylin and eosin stain, ×200).

Our patient had an uneventful post-operative course with no complications. Post-operative prophylaxis of intravenous antibiotics, consisting of a first-generation cephalosporin (cefazolin, 1g every eight hours) and an aminoglycoside (gentamycin 5mg/kg/day) were continued 48 hours after the surgery and low-molecular-weight heparin prophylaxis was administered for 10 days. Active and passive hip ROM exercises were started on the second day post-operatively with mobilizing on crutches, and he was discharged from hospital on the third post-operative day. Full weight bearing without support was allowed at the third post-operative week. The histopathological results of tissue samples were found to be consistent with an osteoarthritic cyst that contained degenerative calcified and necrotic chondroid tissue and bone trabeculae (Figure 3).

There were no complications such as infection and skin necrosis during the follow-up period. At the final follow-up (4 months post-operatively), our patient was assessed clinically. The active ROM for both hips were restricted as had been the case pre-operatively. However, he had only slight pain in his right hip and was able to walk without support. Primary total hip replacement surgery for both hips was recommended in view of the radiological findings.

## Discussion

Tumors and tumor-like lesions located in the pelvis have some specific differences from those tumors arising in other parts of the body. The main difference is that pelvic tumors are located deep in the body, while tumors located in the extremities are relatively superficial. Patients with pelvic tumors are usually older and their tumors larger relative to patients diagnosed as having tumors in the extremities. The clinical presentation is therefore often several years later compared to the same tumor type located in an extremity [12]. The number of benign and malignant soft tissue tumors is much larger than the number of osseous lesions in the pelvic region. However, synovial diseases such as osteoarthritis, rheumatoid arthritis and pigmented villonodular synovitis are common in the hip joint and large subchondral cysts occurring primarily or secondarily to these diseases may raise suspicion of a neoplasm on radiological examination. They can cause massive osseous destruction simultaneously in the femur and acetabulum [12].

Subchondral cysts in degenerative osteoarthritis are often multiple and variable in size, whereas solitary and large cysts are unusual. It has been shown previously that nearly two-thirds of osteoarthritic cysts completely disappear radiographically or become smaller with no additional treatment [13]. However, it is also possible for an osteoarthritic cyst to progress with time if a pathway to the joint space exists.

Some theories have been described to explain the pathophysiology of subchondral cysts in OA. Intrusion of synovial fluid through the articular cartilage secondary to elevated pressure of intra-articular fluid, which results in hydraulic destruction of subchondral bone, has been proposed as a possible etiology [4]. This theory is supported by the presence of defects in the articular cartilage over cysts, of fragments of articular cartilage within cysts, and the similarity of cyst fluid to synovial fluid. However, this theory cannot explain cases where subchondral cysts are not in continuity with the joint space. An alternative hypothesis is that stress-induced micro-fractures lead to secondary effects such as localized areas of bone necrosis, osteoclast resorption and thus cyst formation, with the articular cartilage being left intact [3,5]. This is based on evidence of bony contusions, trabecular fractures and primary subchondral osteolysis, which may, subsequently, communicate with the joint if the overlying articular cartilage and subchondral bone plate cracks.

## Conclusions

Our patient’s case illustrates how an osteoarthritic subchondral cyst can grow into the soft tissue planes in the presence of destruction of the articular cartilage and subchondral bone continuity, and present as a soft tissue tumor.

## Consent

Written informed consent was obtained from the patient for publication of this case report and any accompanying images. A copy of the written consent is available for review by the Editor-in-Chief of this journal.

## Competing interests

The authors declare that they have no competing interests.

## Authors’ contributions

MG and TÖ contributed to the conception and design, and carried out the literature research, manuscript preparation and manuscript review. TÖ, OK and FÖ were involved in the literature review and helped draft part of the manuscript. FA contributed to the conception and design. MG, TÖ and OK revised the manuscript. All authors read and approved the final manuscript.
